# Registered report protocol: domestic violence and mental disorders: gender differences and cumulative effects in a Norwegian population

**DOI:** 10.3389/fpsyt.2025.1531033

**Published:** 2025-04-16

**Authors:** Anne Reneflot, Melanie Straiton, Kim Stene-Larsen, Ann Kristin Knudsen, Benedicte Kirkøen, Ingri Myklestad

**Affiliations:** ^1^ Department of Mental Health, Norwegian Institute of Public Health, Oslo, Norway; ^2^ Centre for Disease Burden, Norwegian Institute of Public Health, Bergen, Norway

**Keywords:** domestic violence, gender differences, mental disorder, diagnostic interviews, registry data

## Abstract

**Introduction:**

Domestic violence (DV) is a significant public health problem linked to poor mental health outcomes. Prior research has relied on self-reported symptoms, overlooked mental disorders prevalent in men, inadequately controlled for confounders, and failed to differentiate between forms and severity of DV.

**Methods:**

This registered report protocol outlines the rationale, design, and planned analyses for investigating the relationship between DV and mental disorders in both Norwegian men and women. We will use data from a population-representative psychiatric diagnostic interview survey linked to national health registries. Key confounders, including sociodemographic and health-related variables, will be adjusted for.

**Results:**

We will examine the association between DV and mental disorders in both men and women, assess cumulative effects of multiple forms of violence, and examine the temporal ordering of DV and mental disorders. Outcomes include anxiety, depression, PTSD, substance use disorder, and suicidality.

**Discussion:**

This study offers population-based insights into the relationship between domestic violence and mental health across genders. The findings may help guide targeted interventions and inform public health policy, particularly in high-income settings.

## 1 Introduction

Domestic violence (DV) is a significant public health issue with severe consequences for those affected (1). Victims of DV face a wide range of negative outcomes including substantial physical, and psychological and social consequences (1–3). DV includes various forms of violence such as physical, sexual, psychological and economic violence. The concept of domestic violence (DV) varies across the literature, with some defining it narrowly as violence between intimate partners and others including violence from other family members. For this study, we adopt the definition outlined in the Istanbul Convention: all acts of physical, sexual, psychological or economic violence that occur within the family or domestic unit or between former or current spouses or partners, whether or not the perpetrator shares or has shared the same residence with the victim (4). However, our study does not include economic violence and we restrict to DV occurring at age 16 and later.

The prevalence of DV varies substantially across countries, study design and populations (5). Intimate partner violence (IPV) is the most commonly reported form of DV (6). Globally, it is estimated that more than one in four women have experienced physical or sexual violence from a partner or ex-partner, with 13 percent experiencing such violence in the past year (7). In Western European countries, the corresponding percentages are one in five and four percent, respectively (7). Fewer studies have examined the prevalence of DV in men. A review examining DV in men found prevalence rates of IPV ranging from three to 20 percent for physical violence and from zero to seven percent for sexual violence (8). However, the reference periods varied across the included studies. Several risk factors are associated with DV, some of the most prominent are gender, low education, living in poverty, alcohol and drug misuse, childhood abuse, and relationship discord (1, 9).

While both men and women experience DV; there are gender differences in both prevalence and severity. Women are more likely to experience severe DV and to experience repeated episodes of DV than men (5, 10, 11). A recent Norwegian study reported lifetime prevalence’s of various forms of IPV (10). The prevalence of less severe violence was 15 percent for men and 13 percent for women, severe violence was three percent for men and 11 percent for women, rape was three percent for men and five percent for women, and the co-occurrence of severe violence and rape was zero percent for men and three percent for women (10). This is in line with other studies finding that women are more likely to experience severe physical violence than men (12, 13). Yet, these studies also show that men are not exempt from being the victim of DV, highlighting the need to study DV in both women and men.

A growing body of research has examined the relationship between DV and mental disorders including anxiety, depression, substance use disorders, post-traumatic stress disorder, self-harm, and suicidality (2, 3, 14, 15). Evidence suggest that DV increased the risk of developing mental disorders, with the strongest associations observed for PTSD, depression and anxiety (15). All forms of DV – physical, psychological violence, and sexual violence – are associated with mental disorders, and victims of DV may experience more persistent mental health problems than non-victims (16–18). Different forms of DV often co-occur (1), with cumulative exposure, especially to sexual abuse, linked to particularly high risk of developing mental disorders (1, 19).

Evidence is inconsistent on whether the link between DV and mental disorders differs by gender. Some studies suggest stronger associations between DV and mental disorders in women than men, while other find no differences (11, 20). This inconsistency can partly be attributed to methodological limitations in the existing literature. Many studies fail to account for severity of DV, focus exclusively on women or on disorders more common among women (e.g. depression) (21–23).

Although the association between DV and mental disorders are firmly documented, the evidence of a causal relationship remains uncertain (1). Studies suggest a bidirectional relationship, where both individuals with a mental disorder have an increased risk of experiencing DV (24, 25), while exposure to DV increases the risk of mental disorders (2, 3, 14, 15). Further, DV and mental disorders share common risk factors that can both confound and moderate the relationship between them (1, 9, 26). For instance, childhood abuse could compound the experience of DV in adulthood, increasing the risk of mental disorders in general and of more severe and enduring disorders in particular (1).

Despite growing research interest, important gaps remain in the literature. Many studies rely on self-reported symptom measures, focus primarily on women, and overlook mental disorders more prevalent among men (1, 8). There are also insufficient control for important confounding factors, and lack of longitudinal, population-representative data (1). Additionally, most studies include one or two types of mental disorders and do not differentiate between the various forms of DV. Incorporating a broader range of disorders, particularly of a diagnostic nature (through interviews or disorders identified by health care professionals) would allow for a comprehensive understanding of the spectrum of mental health issues and the identification of co-occurring disorders.

### 1.1 Objectives and hypotheses

This registered report protocol outlines the methodology and analyses planned to investigate the mental health consequences of DV for both men and women. The proposed study will utilize population-representative data from PsykHUNT, a diagnostic psychiatric interview survey, combined with information from national health registries. PsykHUNT provides information on the onset and of both DV and mental disorders, enabling assumptions about event ordering and detailed analysis of mental disorder persistence in DV victims.

The overall aim is to examine the relationship between DV and mental disorders in both men and women living in Norway. We hypothesize that:

Hypothesis 1 (H1): Both men and women who have experienced DV have a higher risk of developing mental disorders compared to those who have not experienced DV.
Hypothesis 2 (H2): Multiple forms of DV significantly increase the risk of developing mental disorders in both men and women, with cumulative effects observed.
Hypothesis 3 (H3): Experiencing DV causally contributes to the development of mental disorders in both men and women.

### 1.2 User involvement

User involvement is central to this study, contributing helping us align the research questions and methods with real-world needs. The user group includes representatives from organizations focused on violence against both men and women, as well as representatives from the police who work on cases related to domestic violence. They will provide feedback during analysis and actively engage in discussing results and implications, ensuring findings are relevant and actionable for those affected by domestic violence and public health stakeholders.

## 2 Methodology

The registered report protocol complies with the STROBE statement. The survey is registered at ClinicalTrials.gov (identifier: NCT04661228). The survey was a collaboration between the Norwegian Institute of Public Health (NIPH) and the HUNT Research Centre. The project is approved by the Regional Committee for Medical Research Ethics (2017/28/REK midt).

### 2.1 Setting

The study is conducted in Norway, a country with a universal health care system, high level of gender equality, a comprehensive welfare state and a strong legal framework for addressing domestic violence (DV). Norway has implemented various preventive measures, including crisis shelters, and specialized police units. Despite these efforts, DV remains a significant public health concern. The data originate from the county of Trøndelag, which includes both rural and urban centers, including Trondheim, Norway’s third largest city

### 2.2 Procedure

The study uses data from the PsykHUNT study, a population-based psychiatric interview study conducted in the Norwegian county of Trøndelag (27). Participants were randomly selected from individuals who took part in the fourth wave of the Health Survey in Trøndelag (HUNT4) and had consented to be invited to additional studies. HUNT is population-based health survey conducted approximately every 10 years and invites all adults living in Trøndelag to participate (28).

**Figure 1:**
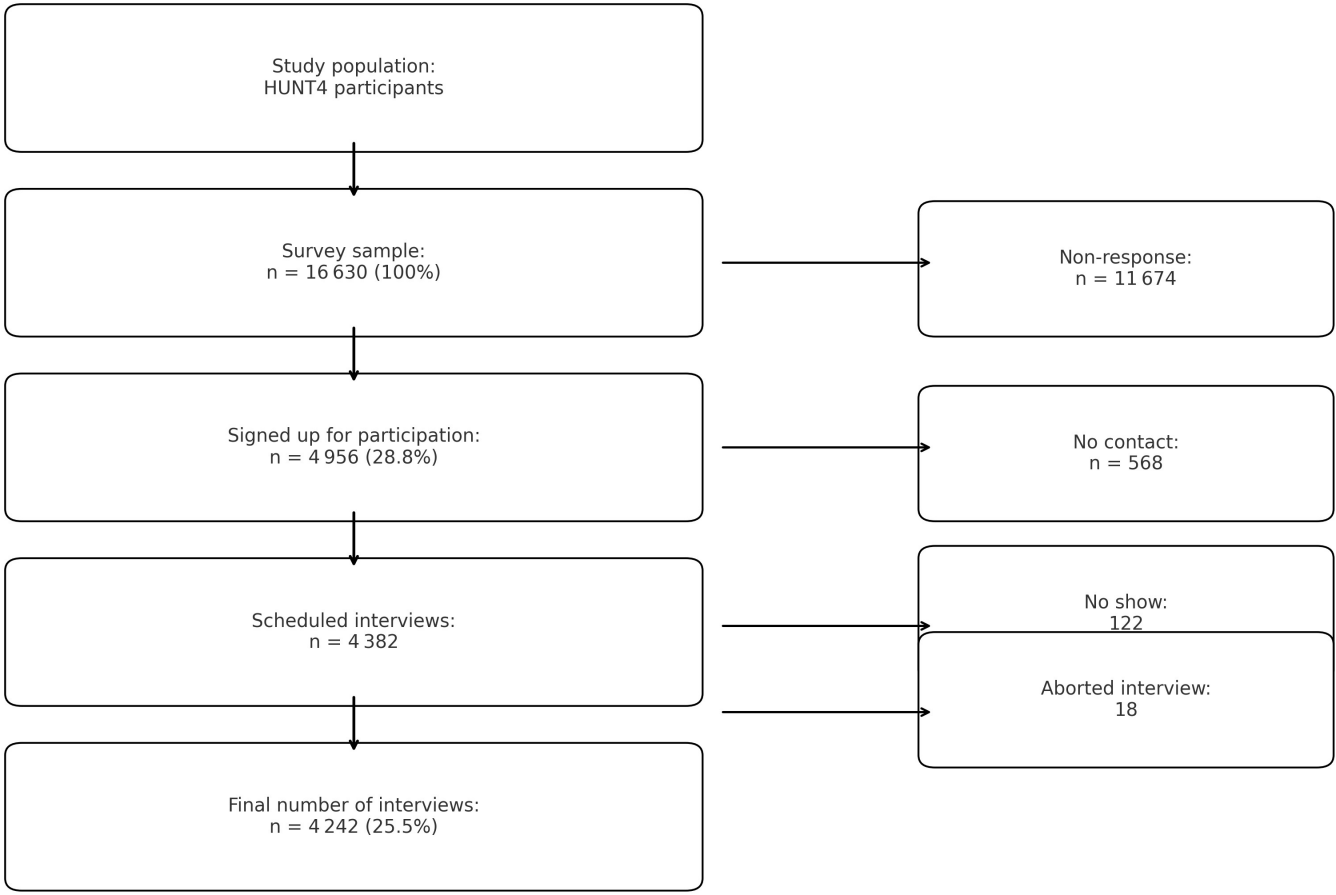
Flow-chart of survey participation process.

The PsykHUNT sample includes 4250 individuals (1 697 men and 2 545 women) aged 20 to 65 years at the time of their HUNT4 participation. Eligible participants had to reside in Nord-Trøndelag or Trondheim and fall within the specified age range. Participants were contacted through postal invitations containing detailed information about the study’s objectives, procedures, ethical approval, GDPR compliance, data handling, and rights to withdraw. In Nord-Trøndelag, participants could sign up by returning a response tag, calling the project coordinator, or sending an SMS or email. In Trondheim, participants received a follow-up SMS about a week after the invitation and could sign up by responding to the SMS or emailing the coordinator. Informed consent was obtained upon registration and participation in the interview. The data collection period lasted from November 2018 to September 2019 in Nord-Trøndelag, and from February 2020 to September 2020 in Trondheim. See **Figure 1** for the sampling process.

PsykHUNT sample is not fully representative of the general population of Nord-Trøndelag, Trondheim, and Norway (27). Participants were more often women, younger, and had a higher educational level than the general population. Specifically, participants in Trondheim were younger and had higher educational attainment compared to those in Nord-Trøndelag. Additionally, few participants had a migrant background, limiting the ability to examine DV and mental health outcomes in this subgroup. The study does not include individuals over 65 years, and therefore cannot address DV in older adults.

### 2.3 Instruments

#### 2.3.1 CIDI

The Composite International Diagnostic Interview (CIDI 5.0) is a structured diagnostic interview developed by the World Health Organization (WHO) to assess mental disorders according to DSM-5 and ICD-10 criteria (29). The interview lasts approximately 90 minutes and was conducted face-to-face by trained lay interviewers using computer-assisted personal interviewing (CAPI). Diagnoses are based on validated algorithms developed by the WHO World Mental Health Survey Initiative. Previous studies have demonstrated high reliability and validity when compared with clinical assessments conducted by psychiatrists (29). One of CIDI’s key strengths is its ability to capture age-of-onset information, allowing for an assessment of temporal sequencing between exposure to domestic violence (DV) and the onset of mental disorders.

#### 2.3.2 National registers

PsykHUNT data were linked to national health and demographic registers using the unique personal identification number assigned to all Norwegian residents. This enabled linkage to:

The Norwegian Population Registry (PR): Includes key demographic variables such as age, sex at birth, marital status, household composition, and number of children.

The Norwegian Patient Register (NPR): Provides information on all inpatient and outpatient contacts with specialist health services in Norway. Psychiatric diagnoses are assigned by healthcare professionals and coded using the International Classification of Diseases, 10th Revision (ICD-10) (30). The register enables the identification of mental disorders treated in specialist settings.

The Patient Registry for the Municipalities (KPR): Contains administrative claims data from general practitioners (GPs), emergency room visits, and other primary care providers. Mental health consultations are coded using the International Classification of Primary Care, Second Edition (ICPC-2) (31). This register captures mental health problems presented in primary care, including symptom and disorder codes.

The Norwegian Education Database (ED): Contains annual data on highest attained education from Statistics Norway.

Income Registry (IR): Includes information on individual annual income reported for tax purposes.

#### 2.3.3 Variables

##### 2.3.3.1 Exposure variables

Exposure to domestic violence was assessed using a standardized module embedded within the CIDI 5.0 interview that covers stressful life events and includes a dedicated PTSD section. Respondents were asked (yes/no) if they had experienced each of the following forms of violence after age 16: (1) death threats or threats of physical violence, (2) physical assault or being beaten up, (3) stalking (i.e., being followed in a harassing, threatening, or intimidating way), (4) rape (defined as non-consensual intercourse or penetration using a penis, finger, tongue, or object, including when the victim was incapacitated, unconscious, or too young to understand), and (5) sexual abuse (defined as attempted rape or unwanted sexual touching).

Those reporting one or more of these experiences were asked follow-up questions about the number of episodes, age at first and last occurrence, and the relationship to the perpetrator. Response options for perpetrator relationship included: current or former spouse/romantic partner, relative, step-relative, in-law, acquaintance, or stranger. Domestic violence (DV) was defined as violence perpetrated by a spouse, partner, ex-partner, relative, step-relative, or in-law after the age of 16. Violence by acquaintances and strangers was excluded from the DV classification

##### 2.3.3.2 Outcome variables

We include the following mental disorders assessed through the CIDI 5.0: depression (major depressive disorder, bipolar I and II), anxiety disorders (generalized anxiety disorder, panic disorder, specific phobia, agoraphobia, social anxiety disorder), substance use disorders (alcohol and drug use), and post-traumatic stress disorder (PTSD). Diagnoses are operationalized using validated algorithms developed for the World Mental Health Surveys. We also include indicators of suicide attempt, suicide thoughts, and non-suicidal self-harm.

From national registry data, we include two additional outcome variables: (1) primary care contact for mental health problems, defined as at least one consultation with a relevant ICPC-2 P-code recorded in the KUHR database, and (2) specialist mental health care contact, defined as at least one consultation with an ICD-10 F-code recorded in the Norwegian Patient Register (NPR).

##### 2.3.3.3 Control variables

The analyses adjust for key control variables, including gender, age, education, marital status, parental status, income, employment, somatic illness, and childhood exposure to violence. These variables are summarized in **Table 1**, which provides an overview of all exposure, outcome, and control variables and their operational definitions.

**Table 1:** Overview and operationalization of study variables.

Variable Category	Variable Name	Source	Operational Definition
Exposure	Physical violence	CIDI	Yes/No. Reported receiving death threats or being physically attacked/beaten up after age 16
Exposure	Psychological violence	CIDI	Yes/No. Reported being stalked or receiving death threats after age 16
Exposure	Sexual violence	CIDI	Yes/No. Reported rape (non-consensual intercourse/penetration) or sexual abuse (unwanted sexual touching) after age 16
Exposure	Recurrent DV	CIDI	Yes/No. Reported ≥2 episodes of any form of DV
Exposure	Cumulative DV	CIDI	Coded as 0 (no exposure), 1 (one type), 2 (two types), or 3 (all three types: physical, psychological, sexual)
Outcome	Any DSM-5 diagnosis	CIDI	Any 12-month DSM-5 diagnosis from CIDI interview (depression, anxiety, PTSD, SUD, etc.)
Outcome	Specific disorders	CIDI	Binary indicators for: Affective disorders, Anxiety disorders PTSD, Substance Use disorders
Outcome	Suicidal ideation	CIDI	Yes/No. Reported suicidal thoughts or wishes to be dead
Outcome	Suicide attempt	CIDI	Yes/No. Reported purposeful self-harm with at least some intent to die
Outcome	Self-harm (non-suicidal)	CIDI	Yes/No. Reported harming oneself without intent to die
Outcome	Primary care contact	KPR	Yes/No. At least one annual consultation with a mental health diagnosis/symptom (ICPC-2 P-codes)
Outcome	Specialist care contact	NPR	Yes/No. At least one annual consultation for a psychiatric diagnosis (ICD-10 F-codes)
Control	Gender	PR	Male/Female (as recorded at interview); participants with incongruent gender and birth sex excluded
Control	Age group	PR	Coded as 20–29, 30–39, 40–49, 50–65 years
Control	Education	ED	Highest completed education, grouped as: high school or less/higher education
Control	Living with partner	CIDI	Yes/No. Living with a spouse or partner
Control	Has children	PR	Yes/No. At least one child in the household
Control	Income quartile	IR	Annual income categorized into quartiles
Control	Employment status	CIDI	Yes/No. Currently in paid employment
Control	Somatic illness	CIDI	Yes/No. Reports at least one chronic somatic condition
Control	Childhood violence	CIDI	Yes/No. Reported having experienced or witnessed DV before age 16

##### 2.3.3.4 Statistical power

To assess study robustness, we conducted a power analysis focused on the overall association between domestic violence (DV) and mental disorders, analyzed separately by gender. Due to the complexity of our research questions, including multiple DV types power calculations for all combinations were impractical. Based on 4,242 participants (1,697 men and 2,545 women), and assuming a DV prevalence of 13% and a mental disorder prevalence of 20%, the study has >80% power to detect an odds ratio (OR) of 1.6 (α = 0.05). These estimates, derived from a two-sample proportion test in STATA 17.0, indicate sufficient power to detect the primary associations of interest (see **Appendix Table 1**).

##### 2.3.3.5 Analysis

We will examine H1 and H2 by conducting unadjusted and adjusted logistic regression analyses separately for men and women and compare the OR of a 12-month DSM-5 mental disorder in CIDI and the annual OR of being diagnosed with an ICD-10 psychiatric disorder in the primary or specialist health care services in DV-victims compared to non-victims. The adjusted analyses will include controls for age and education.

To examine H3, we will make use of information about the age of onset for DV and the age of onset for mental disorders. Both the age of onset of violence and mental disorders in the CIDI are measured through self-report. We first estimate Kaplan-Meier plot comparing the time to onset of mental disorders between those who have experienced DV and those who have not, separately for men and women. If we have enough power, we can divide by type of DV and different diagnosis groups. Next, we apply Cox regression to model the risk of developing mental disorders after an episode of violence, controlled for possible confounding variables. For a summary design and timeline see **Appendix Tables 2, 3**.

All the analyses will be conducted in STATA 17.0.

### 2.4 Relevance of this study

The findings from this study have broader relevance for other high-income countries with comparable healthcare systems and legal frameworks. By combining diagnostic interview data with national health registry information, the study offers robust evidence on the mental health consequences of domestic violence. Its focus on both men and women, as well as varying forms and severity of violence, supports a more nuanced understanding of how DV affects mental health across diverse populations and can inform prevention and policy efforts internationally.

### 2.5 Limitations

This study has several limitations. First, DV exposure is self-reported and subject to recall or reporting bias. Second, the assessment does not include the perpetrator’s gender or capture experiences unique to same-sex relationships. Third, older adults and individuals with migrant backgrounds are underrepresented. Lastly, while temporal data are included, causal inference remains limited due to potential residual confounding.

## Data Availability

Norwegian data protection regulations and GDPR impose restrictions on sharing of individual participant data. However, researchers may gain access to survey participant data by contacting the publication committee (anne.reneflot@fhi.no). Approval from the Norwegian Regional Committee for Medical and Health Research Ethics (https://helseforskning.etikkom.no) is a pre-requirement for access to the data. The dataset is administrated by the HUNT databank, and guidelines for access to data are found at https://www.ntnu.edu/hunt/data. The study protocol and the informed consent form are available on the two homepages of the project; https://www.ntnu.no/hunt/trondelag/psykisk and https://www.fhi.no/cristin-prosjekter/aktiv/diagnosebasert-undersokelse-psykiske-lidelser-og-ruslidelser/.

## Appendix

**Table 1:** Power analysis.

Men							
Alpha	Power	N	N1_noDV	N2_DV	P1_MD	P2_MD	OR
0.05	0.17	1697	1475	221	0.2	0.23	1.2
0.05	0.49	1697	1475	221	0.2	0.26	1.4
0.05	0.79	1697	1475	221	0.2	0.29	1.6
0.05	0.92	1697	1475	221	0.2	0.31	1.8
0.05	1	1697	1475	221	0.2	0.33	2.0
Women							
0.05	0.24	2545	2213	331	0.2	0.24	1.2
0.05	0.66	2545	2213	331	0.2	0.27	1.4
0.05	0.92	2545	2213	331	0.2	0.30	1.6
0.05	1	2545	2213	331	0.2	0.33	1.8
0.05	1	2545	2213	331	0.2	0.36	2.0

N1_noDV, not exposed to DV; N2_DV, exposed to violence; P1_MD, the percentage with a mental disorder among the non-exposed; P2_MD, the percentage with a mental disorder among the exposed

**Table 2:** Study design summary table.

Component	Description
Research Question	What are the mental health consequences of domestic violence (DV) for men and women, and how do different types and severity of DV relate to mental disorders?
Hypotheses	**H1**: Both men and women who have experienced DV have a higher risk of developing mental disorders compared to those who have not experienced DV. **H2**: Multiple forms of DV significantly increase the risk of developing mental disorders in both men and women, with cumulative effects observed. **H3**: Experiencing DV causally contributes to the development of mental disorders in both men and women.
Sampling Plan	**Study Population**: Population-based sample from the PsykHUNT study (part of HUNT4, Norway). **Sample Size**: 4,250 participants (1,697 men and 2,545 women), aged 20-65 years. **Eligibility Criteria**: Participants from Nord-Trøndelag and Trondheim who consented to participation in PsykHUNT. **Recruitment Process**: Random selection from HUNT4 participants, invitation via postal mail and SMS, consent obtained at interview.
Analyses	**H1 & H2:** Logistic regression (unadjusted and adjusted), separate models for men and women. **H3:** Kaplan-Meier survival analysis & Cox regression (age of onset analysis). Sensitivity analyses will be conducted to test robustness of findings.

**Table 3:** Study timeline (Estimated Duration: 18–21 Months).

Phase	Task	Estimated Duration
1. Descriptive & Preliminary Analyses	- Generating descriptive statistics - Checking exposure (DV) and outcome (mental disorders) distributions - Identifying missing data and handling strategies	Months 1–3
2. Main Statistical Analyses	- Conducting logistic regression for H1 & H2 - Running survival analysis (Kaplan-Meier & Cox regression) for H3 - Sensitivity analyses	Months 4–9
3. Writing & Submission of Stage 2 Registered Report	- Finalizing methods and results for Stage 2 submission - Formatting and structuring manuscript per journal requirements - Submitting Stage 2 Registered Report	Months 10–12
4. Journal Review & Revisions	- Responding to reviewer comments (if any) - Revising analyses (if required) and resubmitting - Journal acceptance of final Stage 2 report	Months 13–15
5. Dissemination & Knowledge Translation	- Preparing presentations for conferences - Engaging stakeholders (DV support organizations, policymakers) - Public outreach and media engagement	Months 16–18
